# Pharmaceutical Prospects of Curcuminoids for the Remedy of COVID-19: Truth or Myth

**DOI:** 10.3389/fphar.2022.863082

**Published:** 2022-04-14

**Authors:** Yaw-Syan Fu, Wan-Yi Ho, Ning Kang, May-Jywan Tsai, Jingyi Wu, Liyue Huang, Ching-Feng Weng

**Affiliations:** ^1^ Department of Basic Medical Science, Anatomy and Functional Physiology Section, Xiamen Medical College, Xiamen, China; ^2^ Department of Basic Medical Science, Institute of Respiratory Disease, Xiamen Medical College, Xiamen, China; ^3^ Department of Anatomy, Kaohsiung Medical University, Kaohsiung, Taiwan; ^4^ Department of Otorhinolaryngology, the Second Affiliated Hospital of Xiamen Medical College, Xiamen, China; ^5^ Department of Neurosurgery, Neurological Institute, Neurological Institute, Taipei, Taiwan

**Keywords:** curcumin/curcuminoids, COVID-19, immunomodulation, nutraceuticals, inflammation, antioxidant, chemosensitizing, oxidative stress

## Abstract

Coronavirus disease 2019 (COVID-19) is caused by severe acute respiratory syndrome coronavirus 2 (SARS-CoV-2), which is a positive-strand RNA virus, and has rapidly spread worldwide as a pandemic. The vaccines, repurposed drugs, and specific treatments have led to a surge of novel therapies and guidelines nowadays; however, the epidemic of COVID-19 is not yet fully combated and is still in a vital crisis. In repositioning drugs, natural products are gaining attention because of the large therapeutic window and potent antiviral, immunomodulatory, anti-inflammatory, and antioxidant properties. Of note, the predominant curcumoid extracted from turmeric (*Curcuma longa* L.) including phenolic curcumin influences multiple signaling pathways and has demonstrated to possess anti-inflammatory, antioxidant, antimicrobial, hypoglycemic, wound healing, chemopreventive, chemosensitizing, and radiosensitizing spectrums. In this review, all pieces of current information related to curcumin-used for the treatment and prevention of severe acute respiratory syndrome coronavirus 2 (SARS-CoV-2) infection through *in vitro*, *in vivo*, and *in silico* studies, clinical trials, and new formulation designs are retrieved to re-evaluate the applications based on the pharmaceutical efficacy of clinical therapy and to provide deep insights into knowledge and strategy about the curcumin’s role as an immune booster, inflammatory modulator, and therapeutic agent against COVID-19. Moreover, this study will also afford a favorable application or approach with evidence based on the drug discovery and development, pharmacology, functional foods, and nutraceuticals for effectively fighting the COVID-19 pandemic.

## Introduction

Coronavirus disease 2019 (COVID-19) is an infectious disease that has rapidly spread throughout the world, leading to high mortality rates, and has become a epidemic from the end of 2019. Coronavirus disease (COVID-19), caused by the novel coronavirus—severe acute respiratory syndrome coronavirus 2 (SARS-CoV-2), has surged across the globe, affecting 233 countries or territories, with greater than 337 million confirmed cases and over 5.56 million deaths till January 2022, with the World Health Organization (WHO, 2022) categorizing it as a pandemic (https://covid19.who.int). Infected patients manifest fever, cough, shortness of breath, and lost smell and taste, and critical cases might show acute respiratory infection and multiple organ failure. Probability of these severe indications is further enhanced by age and underlying comorbidities such as diabetes, cardiovascular, or thoracic problems, as well as due to an immunocompromised state (Adhikari et al., 2020). Coronavirus infection, including SARS-CoV, MERS-CoV, and SARS-CoV-2, causes daunting diseases that can be fatal because of lung failure and systemic cytokine storm. The development of coronavirus-evoked pneumonia is associated with excessive inflammatory responses in the lung, releasing extremely high amounts of cytokines known as “cytokine storms,” which result in pulmonary edema, atelectasis, and acute lung injury (ALI) or acute respiratory distress syndrome (ARDS). The pathophysiology of COVID-19 involves the activation of three main pathways: inflammatory, coagulation, and bradykinin cascades (Wiersinga et al., 2020).

SARS-CoV-2 is an enveloped virus belonging to the order Nidovirales, composed of a single-strand, non-segmented, and positive sense RNA genome. SARS-CoV-2 causes COVID-19 that is classified as beta-coronavirus, which primarily occurred and was recorded in December 2019 at Wuhan, China (Godeau et al., 2021). As one member of human coronavirus (HCoV) that has the largest RNA virus genome, the single positive-strand RNA genome of SARS-CoV-2 is approximately 30 kd with two untranslated regions (UTR) linked to the 3′-poly-A tail, and 5′-cap structure, which are crucial for gene transcription and RNA replication. The SARS-CoV-2 genomic structure comprises 5′UTR/NSP/S/3a/E/M/6/7a/7b/8/N/10/3′UTR organization. The non-structural protein (nsp) region of SARS-CoV-2 can be coded and translated as the non-structural proteins 1–16 (nsp1-16), the structural protein regions including spike protein (S), envelop protein (E), membrane protein (M), nucleoprotein (N), and accessory protein regions 3a, 6, 7a, 7b, 8, and 10. Two-thirds of the SARS-CoV-2 genome from the 5′-end contains two open reading frames (ORF), ORF-1a and ORF-1b, which are coded and translated into two long polypeptides, polyprotein 1a (PP1a) and polyprotein 1b (PP1b). The posttranslated cleavage of PP1a and PP1b by two virus-encoded proteases to form 16 NSPs: nsp1-16. Other one-third of the viral genome contains the distinct ORFs derived to form several single-guide RNAs (sgRNAs) that are translated into structural proteins and accessory proteins (Ciotti et al., 2019).

### The Pathogenesis of SARS-CoV-2 Infection

The pathogenesis of the infection severity of SARS-CoV-2 encompasses several stages, the suppression of host antiviral and innate immune responses to increase the infected host cells (Shi et al., 2020; Oh and Shin, 2021), the viral replication-induced infected cell oxidative stress (Laforge et al., 2020; Saheb Sharif-Askari et al., 2020), and injury of infected cells expressing and secreting large amounts of various chemokines or cytokines, colony-stimulating factors, interferons (IFNs), interleukins (IL-1, IL-6, IL-8, IL-12), and tumor necrosis factor-α (TNF-α), which cause acute inflammation described as the “cytokine storm symptom” (CSS) (Junqueira et al., 2021; Kunnumakkara et al., 2021). The CSS of serious SARS-CoV-2 infection causes acute lung injury, tissue fibrosis, and pneumonia. Subsequently, CSS with hemophagocytic lymphohistiocytosis (HLH)/macrophage activation syndrome (MAS) may cause serious systemic hyper-inflammation, plasma leakage, peripheral tissue fluid accumulation, and hypotension. The serious pulmonary inflammation, edema, and tissue fluid accumulation in the lung such as ARDS, combined ARDS, HLH, and MAS will affect gas exchange that leads to systemic hypoxemia and multi-organ failure with disseminated intravascular coagulation (DIC) that have resulted in extreme morbidity and mortality (Horowitz and Freeman 2020; Kunnumakkara et al., 2021; Saad and Moussa 2021). Deficiency in red blood cells, serum, and alveolar glutathione levels has been published in the medical literature for ARDS, as well as viral and bacterial pneumonias, resulting from the increased levels of free radical/oxidative stress (Horowitz and Freeman 2020). Even the patients can recover from ARDS of SARS-CoV-2-infected pneumonia, the lung tissue remodeling and pulmonary fibrosis will continue to last and subsequently limit the pulmonary functional recovery (Bazdyrev et al., 2021; Giacomelli et al., 2021). Additionally, the pathophysiology of COVID-19 is highly heterogeneous, and the way of SARS-CoV-2 modulates the different systems of the host remains unidentified, despite recent discoveries such as viral nucleocapsid (N) protein can bind host mRNA to impair host stress response (Nabeel-Shah et al., 2022); viral infection-induced host hypoxia status can modulate ACE2 expression (Prieto-Fernández et al., 2021); and the overexpression of viral nsp9 can reduce the host’s nucleoporin 62 expression to defective nuclear pore complex formation (Makiyama et al., 2021). Remarkably this deadly virus could affect multiple vital organs and systems (blood, lungs, heart, nervous system, and immune system); however, its exact mechanism of pathophysiology remains obscure. Usually depending on the viral load, infected and sick people commonly manifest fever, cough, shortness of breath, coagulopathy, cardiac abnormalities, fatigue, and death. This complex and multifactorial response of COVID-19 requires a comprehensive therapeutic approach, enabling the integration and refinement of therapeutic responses of a given single compound that has several action potentials. With comprehensive interaction (synergism), biosafety of multi-compounds or multiple treatments need to be taken into consideration and can also provide a promising strategy to cure COVID-19 infection. Currently, several available vaccines and drugs are in the process of evaluation of efficacy and safety, and the determination of dosage in the COVID-19 pandemic. Unfortunately, vaccines are on the market from Pfizer (BNT), Moderna, and AstraZeneca (AZ) with limited supply under an Emergency Use Authorization (EUA) by the WHO. In fact, several vaccines filed phase III clinical trials are still underway from other manufacturers (Srivastava et al., 2021). Intensely, BNT and Moderna vaccines have been currently approved by the FDA (Chilamakuri and Agarwal 2021). Within 2 years, there are several mutated variants of SARS-CoV-2 from the origin strain, and their wide dispersion led to multiple waves of outbreaks, especially the mutation on the spike gene of new SARS-CoV-2 variants caused the alteration in several amino acid residues and change the structural conformation of spike protein, decreasing the titer of human antibodies and neutralizing antibodies induced by infection or vaccination (Khateeb et al., 2022; Ma et al., 2022; Tay et al., 2022). New antibody-resistant variants of SARS-CoV-2 in vaccine breakthrough infection can be seen even in people who have received two or three vaccinations within 6 months which shows that the vaccines have failed to fully protect the people from variants of SARS-CoV-2 infection such as Omicron (Khateeb et al., 2022; Servellita et al., 2022; Wang et al., 2022). On the other hand, exploring the repurposing of natural compounds and drugs may provide an alternative approach or strategy against COVID-19. Various repurposing phytochemicals are showing a broad range of antiviral activities, and its different modes of action have been identified (Kumar Verma et al., 2021; Kumar et al., 2022). Repurposing drugs such as Arbidol, hydroxychloroquine, chloroquine, lopinavir, favipiravir, remdesivir, hexamethylene amiloride, dexamethasone, tocilizumab, and INF-β that neutralize antibodies exhibit *in vitro* anti-coronaviral properties by inhibiting multiple processes in the virus life cycle. Plant-based antiviral compounds such as baicalin, calanolides, curcumin, oxymatrine, matrine, and resveratrol exhibit different modes of action against a wide range of positive-/negative-sense RNA/DNA virus, and future research needs to be conducted to ascertain their role and use in managing SARS-CoV-2 (Rai et al., 2021). Recently, several nutraceuticals have been proven to have an ability of immune-boosting, antiviral, antioxidant, and anti-inflammatory effects in COVID-19 infection (Adhikari et al., 2021; Isidoro et al., 2022), and this scenario will be addressed in the next section.

### Prospective of Natural Compounds on COVID-19 Treatment

Remarkably, using herbal natural compounds is explored as a complementary approach to treating various diseases including COVID-19. Using the active constituents of medicinal plants has long been a well-accepted therapeutic treatment strategy, although understanding their complex pharmacological actions is a major challenge as they provide tremendous chemical varieties and frequently exhibit multi-pharmacological functions (Catanzaro et al., 2018). One report has demonstrated that crude extract or pure compounds isolated from several medicinal plants and/or herbs such as *Artemisia annua*, *Agastache rugosa*, *Astragalus membranaceus*, *Cassia alata*, *Ecklonia cava*, *Gymnema sylvestre*, *Glycyrrhizae uralensis*, *Houttuynia cordata*, *Lindera aggregata*, *Lycoris radiata*, *Mollugo cerviana*, *Polygonum multiflorum*, *Pyrrosia lingua*, *Saposhnikoviae divaricata*, and *Tinospora cordifolia* have shown promising inhibitory effects against coronavirus (Adhikari et al., 2021). Moreover, several phytocompounds including acacetin, amentoflavone, allicin, blancoxanthone, curcumin, daidzein, diosmin, epigallocatechin gallate, emodin, hesperidin, herbacetin, hirsutenone, iguesterin, jubanine G, kaempferol, lycorine, pectolinarin, phloroeckol, silvestrol, tanshinone I, taxifolin, rhoifolin, xanthoangelol E, and zingerol isolated from plants could also be considered as potential drug candidates against COVID-19 (Adhikari et al., 2021). In this article, we draw more attention to the ancient active substance, especially curcumin, to exploit all promising mechanisms of the antiviral property or applications in the treatment of COVID-19.

### Literature Search

**FIGURE 1:**
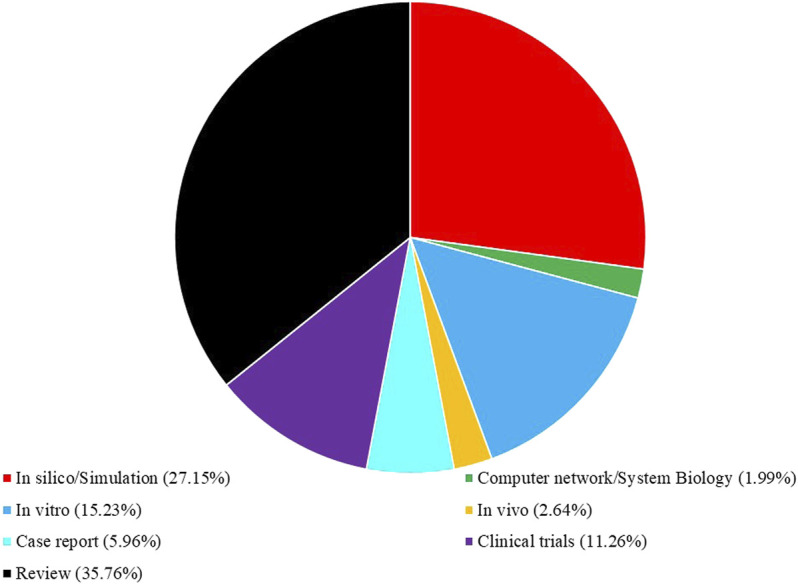
Present proportion (%) of cited literature related to COVID-19 and curcumin according to their categories in this review article.

We reviewed all retracted studies up to Feb 2022 that have evaluated the medicinal benefits of curcumin for COVID-19 infection. The following databases were used: Web of Science, SciVerse, Sci-Hub, Google Scholar, Library Genesis, open access library, Public Library of Science, Scientific Research Publishing, Research Gate, Hindawi, ScienceDirect, Scopus, Medscience, Academia, JAMA, PubMed (NCBI), Springer, Directory of Open Access Journals (DOAJ), Elsevier, Wiley, Taylor and Francis, ProQuest, EBSCO, MEDLINE, and SciELO. Thus, case reports, clinical trials, original research, and review articles were also evaluated. We used free text and Medical Subject Heading (MeSH) terms “curcumin,” “COVID-19,” “ACE2,” “Immunomodulation,” “*In silico*,” “*In vitro*,” “*In vivo*,” and “clinical trial.” The search was done with no language restrictions. All cited articles related to COVID-19 and curcumin were categorized according to MeSH or keywords; the portion of each presenting item in this article is shown in Figure 1.

### Data Extraction After Search

Data extraction was performed according to the following inclusion and exclusion criteria for the current study: studies examining the effects of curcumin on COVID-19 infection were included in this study. We also considered publications in English language including only English abstract. The authors independently obtained data from each selected publication including the type of study, study population (location, age, gender, and sample size), curcumin dose and measured outcomes, and study results.

### Pharmaceutical Potential of Curcumin

For centuries, curcumin chemically known as (1,7-bis(4-hydroxy-3-methoxyphenyl)-1,6-heptadiene-3,5-dione) or diferuloylmethane, is a yellowish pigment and a phenolic component present in the rhizome of turmeric (*Curcuma longa* Linn) and other Curcuma spp., and has been consumed as a flavoring and therapeutic medicinal natural compound. Turmeric grows primarily in Asian countries, particularly India (Yousefian et al., 2019). Curcumin is a plant of the ginger family Zingiberaceae with the related compounds demethoxycurcumin, bis-demethoxycurcumin, and cyclo-curcumin, which are referred to as curcuminoids (Priyadarsini 2014). In *C. longa*, the crude extract curcuminoid makes up 1–6% of turmeric by weight, distributed in 60–70% curcumin (CUR), 20–27% demethoxycurcumin (DMC), and 10–15% bis-demethoxycurcumin (BDMC) (Nelson et al., 2017), whereas commercially available curcumin contains about 77% in curcuminoids (Esatbeyoglu et al., 2012). Curcuminoids vary in potency, effectiveness, and stability, that is, the relative potency for suppression of TNF-induced nuclear factor kappa B (NF-κB) activation was Cur > DMC > BDMC (Sandur et al., 2007). An extensive spectrum of pharmacological and physiological actions of curcumin according to an effective and safe substance has been previously documented in the literature. Numerous studies have shown that curcumin has a broad variety of medicinal functions, including anti-inflammatory (Ghandadi and Sahebkar 2017; Gorabi et al., 2021), antiangiogenic (Sahebkar 2010; Hewlings and Kalman 2017), antidiabetic (Panahi et al., 2018; Riyaphan et al., 2018; Huang et al., 2019), antimicrobial (Praditya et al., 2019; Zahedipour et al., 2020), and antitumor properties (Thiyagarajan et al., 2013; Lin et al., 2020a; Mohajeri et al., 2020). It has also been proven that curcumin confers the therapeutic benefits in inflammatory disorders, neoplasms, neurodegenerative disorders, rheumatologic diseases, and cardiovascular diseases (CVDs) (Aggarwal and Harikumar 2009; Velmurugan et al., 2018). Of note, there is scientific evidence of a beneficial impact on human health in functional foods produced by incorporating plant parts that have known or unknown bioactive components like flavonoids, phenolic acid, and alkaloids with various biological properties as aforesaid (Tsuda 2018; Xu et al., 2018; Roy et al., 2022). However, curcumin has some limitations in terms of physicochemical properties such as very low aqueous solubility, high degradation, and limited activity only in acidic pH, which leads to a decrease in bioavailability (Wan et al., 2012; Peng et al., 2018; Dei-Cas and Ghidoni 2019). Importantly, curcumin has been demonstrated to impede inflammation, oxidative stress, cancer cell proliferation, and cell death and abrogate infections caused by bacteria, fungi, and viruses (Omosa et al., 2017; Fu et al., 2021). Additionally, curcumin treatment supports total white blood cell counts and increases the levels of antioxidant indicators, circulating levels of antibodies to sheep red blood cells (SRBC), and plaque-forming cells (PFC) in the spleens of mice (Antony et al., 1999; Shakeri et al., 2017; Afolayan et al., 2018). Furthermore, it also enhances the phagocytic action of macrophages in various animal models including mouse (Wang et al., 2016; Sohn et al., 2021), ferret (Huang et al., 2021) and hamster (Lee et al., 2022; Seldeslachts et al., 2022). As mentioned before, curcumin is a polyphenolic compound, and the activity associated with its polyphenolic chemical structure varies depending on its concentration as well as the cell types and their status (sometimes it can act as a reactive oxygen species (ROS) scavenger, sometimes it can increase ROS production and cause apoptosis, etc.) (Balasubramanyam et al., 2003; Kim et al., 2016; Lin S.-R. et al., 2017; Larasati et al., 2018). From a molecular point of view, curcumin may mediate its pharmacological activities through Janus kinase/signal transducers and activators of transcription (JAK-STAT) (Rajasingh et al., 2006; Salehi et al., 2019), NF-κB (Marquardt et al., 2015; Mortezaee et al., 2019), protein kinase B (AKT or PKB) (Wang et al., 2019), transforming growth factor β (TGF-β) (Li et al., 2013; Thacker and Karunagaran 2015), and mammalian target of rapamycin (mTOR) (Lin J. et al., 2017; Thiyagarajan et al., 2018). In particular, the transcription factors nuclear factor E2-related factor 2 (Nrf2) and NF-κB can regulate 1) the inhibition of the transcription factor NF-κB mediates anti-inflammation, and 2) the induction of Nrf2 signaling pathways promotes antioxidant defense mechanisms and production of phase II enzymes (Dai et al., 2018; Esatbeyoglu et al., 2012). Anticarcinogenic effects of curcumin are also related to an increase in the p53 levels and thus in pro-apoptotic Bax and cytochrome C. The suppression of proliferation and a cell cycle arrest can also be modulated by curcumin through a p53-independent pathway such as the inhibition of NF-κB inhibitor α (IκBα), B-cell lymphoma 2 (Bcl-2), B-cell lymphoma-extra large (Bcl-xl), cyclin D1, and IL-6. Moreover, apoptosis can be initiated by curcumin by the increased cleavage of poly (ADP-ribose) polymerase (PARP) (Xu and Liu 2017; Esatbeyoglu et al., 2012). *In vitro* and *in vivo* studies of influenza A virus infection have shown curcumin treatment could suppress influenza A virus absorption and replication by activation of the Nrf2 pathway to suppress the NF-κB pathway and inflammatory cytokines to attenuate the oxidative stress and symptoms (Dai et al., 2018).

### Challenges of Curcumin on COVID-19 Treatment

Several lines of evidence suggest curcumin as a talented prophylactic and therapeutic candidate for COVID-19 in the clinic and public health settings. First, curcumin exerts antiviral activity against many types of enveloped viruses, including SARS-CoV-2, through multiple mechanisms: direct interaction with viral membrane proteins, disruption of the viral envelope; inhibition of viral proteases, and induction of host antiviral responses. Second, curcumin protects from lethal pneumonia and ARDS *via* targeting NF-κB, inflammasomes, IL-6 trans-signal, and high-mobility group box 1 (HMGB1) pathways. Third, curcumin is basically recognized as safe and well-tolerated in both healthy and diseased human subjects (Miryan et al., 2021; Thimmulappa et al., 2021).

According to the aforementioned understanding about the COVID-19 pandemic and pharmacological merits of curcumin, the therapeutic use in SARS-CoV-2 infection remains to be explored. In this context, curcumin/curcuminoids have been shown to possess beneficial effects on the progression of inflammatory diseases including COVID-19 based on numerous action mechanisms including antiviral, anti-inflammatory, anticoagulant, antiplatelet, and cytoprotective (Babaei et al., 2020; Rattis et al., 2021). These investigations and many other effects of curcumin make it a promising agent in the adjuvant treatment of COVID-19; however, all current entities related to curcumin used for proposed treatment and prevention of COVID-19 infection are mostly based on *in vitro*, *in vivo*, *and in silico* studies, clinical trials, and new formulation designs. In fact, there is no direct use in the SARS-CoV-2-infected patients, so it is necessary to re-evaluate the efficacy of curcumin and provide deep insights into knowledge and strategy about curcumin’s role as an immune booster, inflammatory modulator, and therapeutic agent against COVID-19.

### Curcumin as the Anti-Inflammatory Drug Candidate and Immune Modulator

There is a great variation in the clinical symptoms of SARS-CoV-2 infection from asymptomatic infection, upper respiratory tract infection, pneumonia, ARDS to multi-organ dysfunction. Although the patients recover from ARDSs of serious COVID-19 pneumonia, their lung parenchyma tissue remodeling and pulmonary fibrosis will continue to suffer and limit the pulmonary functional recovery of the patients, indicating that anti-inflammation will be one of the most important focuses on the treatment of SARS-CoV-2 infection. As an inflammatory response, NAcht leucine-rich repeat protein 3 (NLRP3) inflammasomes induce the production of several cytokines, which have been confirmed to play major roles in the pathogenesis of several viral diseases, including COVID-19 (Freeman and Swartz 2020; Zhao M. et al., 2021; Junqueira et al., 2021; Pan et al., 2021). The key molecular mediators of inflammation include pro-inflammatory cytokines such as TNF-α; chemokines; inflammatory enzymes such as cyclooxygenase-1, and -2 (COX-1, COX-2), matrix metalloproteinase-9 (MMP-9), and 5-lipoxygenase (5-LOX); transcription factors such as signal transducer and activator of transcription 3 (STAT3) and NF-κB; and ILs, for example, IL-1, IL-6, and IL-8 (Ghasemi et al., 2019; Norooznezhad et al., 2020), and notably the inhibition on those proteins or pathways can be considered as the major therapy targets for anti-inflammatory treatments of COVID-19. On the other hand, the cellular oxidative stress could be attenuated by the Nrf2 and heme oxygenase-1 (HO-1) pathway, which helps in decreasing and maintaining the redox balance in cells thereby reducing inflammation (Yang et al., 2009; Ahmed et al., 2017; Zhang et al., 2021); the activation and increase in Nrf2 expression can suppress and downregulate NLRP3 inflammasomes (Hennig et al., 2018; Yarmohammadi et al., 2021); and the activation of Nrf2 relative pathways can be a strategy to attenuate the infective symptoms of COVID-19. Experimentally, *in vitro* curcumin treatment as an Nrf2 activator can stimulate the Nrf2 signaling pathway to deter the NF-κB pathway and suppress viral replication in the influenza A virus infection with an IC_50_ of 140.67 μg/ml. *In vivo*, rodents treated with curcumin (50 mg/kg) significantly promoted Nrf2 expression to scavenge ROS that protects cells against oxidative stress and lung injury (Dai et al., 2018; Lin and Yao, 2020). In *in vivo* experiments, gavage with curcumin (40–50 μg/ml) inhibited the activation of NLRP3 by triggering the SIRT1/Nrf2 pathway to abrogate the downstream cytokine expression, such as IL-1β, IL-6, IL-18, and TNF-α (Yin et al., 2018; Yin et al., 2020). For virus-induced inflammation, curcumin treatment can revoke pro-inflammatory cytokine production, such as IL-1β, IL-6, and TNF-α via the inhibition on the NF-κB signaling pathway to hinder NLRP3 expression (Sordillo and Helson 2015; Xu and Lin 2017; Saeedi-Boroujeni et al., 2021). Based on the anti-inflammatory dosage of curcumin in rodent experiments and the Guidance for Industry of the FDA (https://www.fda.gov/media/72309/download), we found that the effective dose for human adults (70 kg) is about 450–550 mg/day using the surface area-to-body weight relative relationship.

The anti-inflammation of curcumin suppresses the expressions of inflammatory mediators not only by triggering the SIRT1/Nrf2 pathway but also activating PPARγ, IL-10, and AMPK pathways. Through the activation of IL-10, AMPK, and PPARγ pathways, curcumin can downregulate NF-κB, COX-2, inducible nitric oxide synthase (iNOS) expression, and prostaglandin E2 (PGE2) levels (Zhai et al., 2015; Kumar et al., 2017), and through the activation of the PPARγ pathway, curcumin can obstruct NF-κB, STAT, activator protein 1 (AP-1), and mitogen-activated protein kinases (MAPK) (Carvalho et al., 2021). The inhibition of NF-κB can decrease the expression of several genes, including COX-2, MMP-9, IL-8, iNOS, and TNF-α (Bengmark 2006). Through the activation of the AMPK pathway, curcumin can hinder mTOR, p38, p53, and COX-2 pathway (Thiyagarajan et al., 2018; Soltani et al., 2019), and through IL-10, curcumin can also increase the HO-1 expression and modulate the immune response (Naito et al., 2014). Based on the aforementioned studies, indirect anti-inflammatory investigations including signaling pathways are affected by curcumin, but very few results are directly pertaining to the SARS-CoV-2 treatment, and interestingly, there is still promising beneficial potential for treatment and prevention of COVID-19 in the future.

### *In Vitro* and *in Vivo* Validations of Curcumin Use in SARS-CoV-2 Infection

According to the quick spread and high mortality of COVID-19, several clinical trials of the antiviral drugs or formulations applied for COVID-19 treatment were conducted or recruited without passing the *in vitro* and *in vivo* tests. Only very few *in vitro*-tested models of curcumin for COVID-19 treatment were investigated using Vero E6 cells or human Calu-3 cells that were infected with SARS-CoV-2 (Bormann et al., 2021). The data revealed that *in vitro* models treated with curcumin, nano-curcumin formulation, or turmeric root extracts could neutralize and decrease the SARS-CoV-2 viral RNA level at low subtoxic concentrations (Guijarro-Real et al., 2021; Pourhajibagher et al., 2021). Another biochemical and enzyme activity tests showed that the turmeric root extract can significantly constrain the main protease (M^pro^) activity of SARS-CoV-2 (Dound et al., 2021). Within the past 3 months, the results of a new *in vitro* study by Bahun et al. (2022) showed the IC_50_ of curcumin on the SARS-CoV-2 protease 3CL^pro^ is 11.9 μM, but in another *in vitro* study by Marín-Palma et al. (2021), results illustrated that curcumin has the EC_50_ from 1.14 μg/ml to 6.03 μg/ml in the antiviral effects of several different SARS-CoV-2 variants, whereas the data revealed curcumin has a cytotoxic effects with the CC_50_ about 16.5 μg/ml, which is equal to 6.1 μM. The results of the two *in vitro* studies demonstrate that curcumin has the potential to inhibit the SARS-CoV-2 infection, but its effective and cytotoxicity doses are very close and have some overlapping that needs additional studies to evaluate its practical safe dose and treatment mode of its application for COVID-19 treatment. Due to urgent demands for curing or preventing COVID-19, there were several clinical trials based on the application of curcumin on COVID-19 treatment that are either under recruitment or completed, but only few non-human *in vivo* animal studies that are simulated to apply a curcumin formulation on COVID-19 therapy has been published. One study used human beta-coronavirus to mimic the SARS-CoV-2 infection (Dound and Sehgal 2021), and the result showed the application of a curcumin-based herbal formulation could significantly increase CD4^+^ and CD8^+^ cell count in blood and plasma IgG and IgM levels in virus-infected animals. Mounting evidence obtained from preclinical studies using animal models of lethal pneumonia shows curcumin exerts the protective effects by regulating the expression of both pro- and anti-inflammatory factors such as IL-6, IL-8, IL-10, and COX-2, promoting the apoptosis of polymorphonuclear leukocytes (PMNs) and scavenging ROS which exacerbates the inflammatory response (Liu and Ying, 2020).

### *In Silico* Study of Curcumin on Viral Proteins

There are several major viral site targets for COVID-19 therapy or prevention simulated by *in silico* studies. The molecular docking results revealed that curcumin has multiple targets with potential to bind with five viral proteins of SARS-CoV-2, and those candidate proteins contain S protein, main protease (M^pro^), RNA-dependent RNA polymerase (RdRp, nsp12), nucleic acid-binding protein (nsp9), and RNA uridylate-specific endoribonuclease (nsp15). *In silico* studies showed curcumin can bind and act on the host proteins that participate SARS-CoV-2 infection, such as angiotensin-converting enzyme 2 (ACE2), and several proteins applied for intracellular signal transduction pathways and modulate cytokines secretion (Noor et al., 2021).

There are two domains, S1 and S2, which consist the spike protein of SARS-CoV-2 (Walls et al., 2020), and 319–541 aa of S1 is known as the receptor-binding domain (RBD) (Trigueiro-Louro et al., 2020). Within the RBD of S1, 437–508 aa are known as an ACE2 receptor-binding motif (RBM) (Rath and Kumar 2020). The COVID-19 virus entry into host cells is initiated by binding of S protein to the host cell surface ACE2 as its target receptor (Yan et al., 2020), and the RBD is the virus–host binding spots (Veeramachaneni et al., 2021). *In silico* simulation data exhibited that curcumin has a high binding affinity to the RBD and ACE2 (Babaeekhou et al., 2021; Nag et al., 2021; Umashankar et al., 2021). The binding of amino acid residues to curcumin presented near the RBM of S1 protein, and some curcuminoids have stable interactions with key spot residues for the binding of ACE2 comprising the glycosylation site (Babaeekhou et al., 2021), suggesting the potential efficacy of curcumin/curcuminoids in hindering the formation of S protein–ACE2 complex (Jena et al., 2021). A recent genome sequencing study also indicated that the spread of double mutations at E484Q/L452R, T478K/L452R, and F490S/L452Q of RBD has the latent potential for the enhancement of viral mutated S protein (S^m^) and host ACE2 to form S^m^–ACE2 binding (Aggarwal et al., 2021), and concurrently, that may cause more and effective infection of SARS-CoV-2 mutants. Interestingly, the molecular docking results showed that curcumin and piperine have been demonstrated not only high binding potential with native S protein but also similar or more stability of binding potential with S^m^ or/and S^m^–ACE2 complex (Nag et al., 2021).

The nsp12 is the RNA-dependent RNA polymerase (RdRp) of SARS-CoV-2, and its binding with nsp7 and nsp8 to form the nsp12–nsp7–nsp8 complex is the central component for viral replication and transcription (Gao et al., 2020; Peng et al., 2020). The inhibition of RdRp could abrogate SARS-CoV-2 replication; thus, this nsp12–nsp7–nsp8 complex is recognized as a potential target for COVID-19 treatment (Khan et al., 2020; Begum et al., 2021; Ruan et al., 2021). Compared with antiviral drugs (favipiravir and remdesivir), the results of molecular docking and molecular dynamic simulation showed curcumin/curcuminoids have a good binding affinity and stability with RdRp–RNA complex of SARS-CoV-2 (Kumar Verma et al., 2021; Singh et al., 2021) and also showed they have higher potential to be developed as viral replication inhibitors of COVID-19.

The genome of the COVID-19 virus is encoded in two protease enzymes, main protease (3CL^PRO^ or M^pro^-nsp12) and papain-like protease (PL^pro^, nsp3), which are involved in the proteolytic processing viral polyproteins into functional proteins for viral replication and genomic expression within the host cells (Das et al., 2021). The proteolytic cleavage by viral proteases at the posttranslational stage plays the crucial role in the life cycle of SARS-CoV-2. M^pro^ as a main protease can catalyze more than 11 proteolytic cleavage sites for nsp generation and without human homologs (Dai et al., 2020); therefore, M^pro^ is considered to be an ideal antiviral target for COVID-19 treatment (Li and Kang 2020). Results of *in silico* studies show that curcumin/curcuminoids can form strong bonds with the active site of SARS-CoV-2 M^pro^ (Ibrahim et al., 2020; Bahun et al., 2022). Due to high binding affinity and its binding with the interface region of M^pro^ may cause the protein conformational changes, indicating that curcumin/curcuminoids could be the potential ligands for COVID-19 therapy (Ibrahim et al., 2020; Li and Kang 2020; Kumar M. et al., 2021; Mahmud et al., 2021a; Babaeekhou et al., 2021; Teli et al., 2021; Halder et al., 2022). One more study demonstrated that demethoxycurcumin and bisdemethoxycurcumin had an optimum binding affinity with COVID-19 M^pro^ by molecular modeling and showed the stable state by molecular dynamic (MD) simulation assay, suggesting these could be one of the potential ligands for COVID-19 therapy (Mulu et al., 2021). Unfortunately, there are only two reports with *in vitro* validation of the inhibitory and selectivity effects of curcumin on SARS-CoV-2 infection to confirm its powerful potential on COVID-19 therapy (Kandeil et al., 2021; Bahun et al., 2022).

The nsp9 is single-strand RNA-binding protein of SARS-CoV (nsp9–SARS) which was found to be essential for viral replication (Frieman et al., 2012). nsp9 of SARS-CoV-2 can bind to the nidovirus RdRp-associated nucleotidyltransferase (NiRAN) domain relating the viral replication and transcription (Kumar M. et al., 2021), and it was also considered to be the therapy target for COVID-19 treatment (Zhang et al., 2020; Kumar et al., 2021b). There were several studies to screen the potential candidate compounds to bind or act on nsp9 by *in silico* tools (Barros et al., 2020; Chandra et al., 2021; Junior et al., 2021), and one *in vitro* cellular assay showed the inhibition on nsp9 can reduce the viral replication of SARS-CoV-2 (Littler et al., 2021). The molecular docking results showed that curcumin can bind to the ligand binding site of nsp9, and curcumin can form about 11 interaction sites with nsp9 (Kumar et al., 2021b).

The nsp15 of SARS-CoV-2 is a RNA uridylate-specific endoribonuclease (NendoU) and a conserved protein in coronavirus (Bai et al., 2021). nsp15 can degrade negative-strand viral RNA to protect virus from the host immune responses (Hackbart et al., 2020; Zhao et al., 2020; Gao et al., 2021), and the inhibition of nsp15 can also promote viral elimination by the host immune system (Kumar et al., 2021c). There were several *in silico*-based studies on nsp15 to find potential antiviral compounds or candidates (Mahmud et al., 2021b; Canal et al., 2021; Quimque et al., 2021; Zrieq et al., 2021), and only few studies have conducted further validation of the antiviral effects by *in vitro* or *in vivo* study (Canal et al., 2021; Kumar et al., 2021c). The molecular docking result showed curcumin can bind to nsp15, which might have the potential to inhibit SARS-CoV-2 replication (Kumar et al., 2021d).

Gaining deep insights into all those viral protein *in silico* studies of SARS-CoV-2, we infer that curcumin can bind and interact with several target viral proteins that assist in viral attachment (S protein), replication (nsp12, nsp9), posttranslational protein cleavage/modification (M^pro^), and host immunity evasion (nsp15), suggesting that curcumin/curminoids provide a promising hit against COVID-19 even without any additional demonstration (*in vitro* and *in vivo* validation). To meet the urgent demand for managing the COVID-19 pandemic, curcumin use will be the first priority with high biosafety because the toxicity of curcumin is very low even in high amounts up to 12–18 g/day (Fu et al., 2021).

### *In Silico* Study of Curcumin on Host Proteins Associated With COVID-19 Infection

As SARS-CoV-2 is a single-strand RNA virus, its mutation may quickly and easily evade the host immune system or targeting drugs; therefore, targeting on the inhibition of host proteins that participate in the viral infections will have the stable therapeutic advantages for COVID-19 treatment or prevention. The viral infection of SARS-CoV-2 is initiated by its S protein binding to ACE2 of the host cellular surface (Alcocer-Díaz-Barreiro et al., 2020; Jahanafrooz et al., 2022). Several *in silico* studies showed curcumin can bind to the S protein and ACE2 to restrict viral entry (Maurya et al., 2020; Nag et al., 2021). The molecular docking results showed that curcumin not only exhibits high interaction with ACE2 but also has the most potent binding with S^m^ and S^m^–ACE2 complex (Nag et al., 2021), indicating that curcumin can be applied in treatments for different SARS-CoV-2 mutated strand infections. The human serine protease serine 2 (TMPRSS2) is a transmembrane protease of host cells; it cleaves and activates the S protein of SARS-CoV-2 to bind with ACE2 on the initiated stage of the viral infection (Singh et al., 2020) and reveals that TMPRSS2 is a suitable target for COVID-19 therapy. TMPRSS2 genes may be co-expressed with SARS-CoV-2 cell receptor genes ACE2 and basigin (BSG), but only TMPRSS2 is demonstrated to have tissue-specific expression in alveolar cells (Brooke and Prischi 2020). Acetaminophen (paracetamol) and curcumin can downregulate the expression of TMPRSS2 in human cells (Zarubin et al., 2020). In addition to the major mode of viral infection by S protein binding to ACE2, SARS-CoV-2 can infect host cells by endocytosis and follow the proteolytic activation by cathepsin L (Jackson et al., 2021; Takeda 2021). Based on the MD simulations, curcumin was found to be the inhibitor of TMPRSS2 (Umadevi et al., 2020; Jackson et al., 2021), and *in vitro* results showed curcumin treatment can also impede the activity of cathepsin L (Goc et al., 2021; Oso et al., 2022). Another key host cell membrane protein in SARS-CoV-2 infection is the glucose-regulating protein 78 (GRP78) receptor. GRP78, also termed as a HSP5A or binding immunoglobulin protein (Bip), is one member of the 70-kDa heat shock protein (HSP70) family and functions as the endoplasmic reticulum (ER)-resident molecular chaperone for the clearance of misfolded proteins (Dessie and Malik 2021). An intracellular ER stress increase may upregulate the GRP78 expression and induce GRP78 re-localization to the cell membrane as cell surface GRP78 (Elfiky et al., 2021). The cellular surface GRP78 can associate with the major histocompatibility complex class I (MHC-I) that may aid SARS-CoV-2 to get into the host cells for starting an infection or help viral release from infected host cells (Ha et al., 2020; Gonzalez-Gronow et al., 2021). *In silico* data showed curcumin could interact with the S protein binding site and ATPase domain of GRP78 (Allam et al., 2020; Sudeep et al., 2020), suggesting that curcumin can assist in preventing COVID-19 viral attachment and enter host cells by binding to and inhibiting GRP78 of host cell surface.

*In silico* studies of curcumin/curcuminoids on the viral proteins and host proteins with SARS-CoV-2 infection are given in Table 1. It was deems the same as the viral site; all *in silico* results of the host proteins on SARS-CoV-2 infection show that curcumin can bind and interact with several targeting host proteins that play roles in viral binding (ACE2) and S protein activation (TMPRSS2, cathepsin L), and aid viral entry host cells (GRP78). Multiple targeting effects of curcumin on the site of virus and host cells during SARS-CoV-2 infective stages show its high potential to be applied for COVID-19 prevention or therapy, but until now, validations by *in vitro* or/and *in vivo* studies are still few or nil, and strikingly, it is only applicable as a co-supplement or adjuvant for therapy; nowadays, filing for approval by the FDA to become a real anti-COVID-19 medicine is most challenging.

**TABLE 1:** *In silico* studies of curcumin/curcuminoids on the viral proteins and host proteins with SARS-CoV-2 infection.

	Protein	Description/functions	*In silico* binding sites	References
**Viral proteins**	nsp 12	RNA-dependent RNA polymerase, virus RNA replication	Curcumin: Lys545, Arg553, Ser759, Ser682, Arg555, Ala688, Val557dDiacetylcurcumin: Thr680, Asn691, Thr687, Lys545, Arg555, Asp623, Val557, Asp761	PDB ID: 7BV2 (Singh et al., 2021)
			Curcumin: Asn691, Asp623, Arg624	PDB ID: 6M71 (Kumar et al., 2021d)
	S protein	Receptor-binding domain (RBD) of spike protein	O-Demethyl demethoxycurcumin: Cys336, Asp364, Leu335, Phe338, Asp364, Val367, Leu368, Phe374, Phe374, Trp436	PDB ID: 6VSB (Umashankar et al., 2021)
			Curcumin: Arg328, Pro527, Lys529, Asn542, Ser98, Asn121, Arg190, Ser730, eu861, Asp867, Phe970, Asp994, The998	PDB ID: 6VSB (Babaeekhou et al., 2021)
			Curcumin: Leu452, Glu484, Phe490, Ser494, Tyr495 for S; Arg403, Try449, Leu452, Phe453, Lys484, Asp494 for Sm	PDB ID: 6M0J (Nag et al., 2021)
			Curcumin: Leu546, Gly548, Phe541, Asn856, Leu997, Ser967, Asp571, Ala570, Val976, Thr572, Asp979, Thr547, Arg1000, Ser975, Thr573, Asn978, Cys743, Thr573, Asn978, Cys743, Leu966	PDB ID: 6VSB (Jena et al., 2021)
			Curcumin: Tyr505, Ala387, Asp38, Gln493, Glu 35, His34, Glu 37, Arg393	PDB ID: 6VW1 (Kumar Verma et al., 2021)
	Mpro	Main protease, 3-chymotrypsin-like cysteine protease (3CLpro), there are 11 proteolytic cleavage sites of Mpro on the posttranslation of viral gene expressions	Curcumin: His41, Leu141, Asn142, Glu166, Gln189	PDB ID: 6M03 (Babaeekhou et al., 2021)
			Curcumin: Thr190, Pro168, Met165, Glu166, Cys145	PDB ID: 6LU7 (Kumar Verma et al., 2021)
			Curcumin: His163, Cys145, Gly143, Ser144, Leu141	PDB ID: 6LU7 (Ibrahim et al., 2020)
			Curcumin: Asn142, Gln192; demethoxycurcumin: Leu272, Thr199, Lys137; and bisdemethoxycurcumin: Phe294, Gln110, Glu240	PDB ID: 7BUY (Mulu et al., 2021)
			Curcumin: Gly143, Gln189, Thr190, Pro168, Leu141, Glu166, Cys145, Met165, Pro168	PDB ID: 6LU7 (Mahmud et al., 2021a)
			Curcumin: Thr26, Gly143, Cys145	PDB ID: 6LU7 (Teli et al., 2021)
			Curcumin: Gly143, Ser144	PDB ID: 6LU7 (Halder et al., 2022)
			Curcumin: Thr26, His41, Gln89	PDB ID: 6LU7 (Kandeil et al., 2021)
			Curcumin: Met49, Met165, Glu166, Arg188, Gln189, Gln192	PDB ID: 6M2N (Bahun et al., 2022)
			Curcumin: Leu141, Gly143, Ser144, Cys145, His163, Met165, Thr190	PDB ID: 6M2N (Adhikari et al., 2022)
			Curcumin: Thr190, Pro168, Met165, Glu166 and Cys145	PDB ID: 6LU7 (Umadevi et al., 2020)
	nsp 9	Nucleic acid-binding protein	Curcumin: Met16, Gly41, Gly42, Arg43, Val45, Phe60, Pro61, Lys62, Ser63, Ile69, Thr71	PDB ID: 6W4B (Kumar et al., 2021a)
	nsp 15	RNA uridylate-specific endoribonuclease (NendoU) activity, degrades viral RNA	n.a.	PDB ID: 6VWW (Kumar et al., 2021d)
**Host proteins**	ACE2	Angiotensin-converting enzyme-2, serve as viral spine protein receptor	Curcumin: Leu591, Lys94, Asn210, Glu564, Glu280, Tyr207, Asp206, Gly205, Tyr196, Ala99, Lys562, Ala396, Gln102, Trp566, Gln98, Val209, Pro565, Val212, Leu95	PDB ID:LR42 (Jena et al., 2021)
			Curcumin: Ser44, Ala46, Ser47, Gly66, Trp69, Ser70, Lys74, Ser77, Glu110, Met62, Leu73	PDB ID: LR42 (Kumar Verma et al., 2021)
			Curcumin: Ala348, His378, Asn394, Tyr385, His401, Glu402	PDB ID: 1R42 (Maurya et al., 2020)
			Curcumin: Ans210, Lys94, Leu91, Ala396, Lys562, Ala99, Try196, Gly205, Try207, Glu208, Glu564, Asp206, Gln102, Trp566, Gln98, Val209, Pro565, Val212, Leu95	PDB ID: n.a. (Kumar et al., 2021f)
	GRP78	Glucose-regulating protein 78 (GRP78) receptor, ER molecular chaperone, and cell surface GRP78 help viral infection	Curcumin: Thr39, Ile61, Glu201, Asp224, Phe258, Glu228	PDB ID: 5E84 (Allam et al., 2020)
			Curcumin: Ile426, Thr428, Thr434, Phe451	PDB ID: 5E84 (Sudeep et al., 2020)
	Cathepsin B/K/L	Host cysteine protease serves as viral spine protein activator	Curcumin: Gly143, Ser144 Cys145, His172	PDB ID: 3KW9 (Oso et al., 2022)
	TMPRSS2	Transmembrane serine protease 2, cleaving and activating S protein of SARS-CoV-2	Curcumin: Gly148, Asp147, Ser195	PDB ID: 2OQ5 (Umadevi et al., 2020)

n.a. non-available.

### Clinical Trials of Curcumin on COVID-19 Treatment

Remarkably, there are numerous reports of *in silico*, *in vitro*, and *in vivo* studies demonstrating that curcumin has the therapeutic potential against the COVID-19 based on its anti-inflammation, antioxidant, and antiviral effects. In this section, we have reviewed many clinical trial applications and published reports regarding the antiviral potential of curcumin/curcuminoids on COVID-19 therapy from PubMed and clinicaltrials.gov. In all these clinical trials, curcumin/curcuminoids formulations are used as a therapeutic co-supplement in the treatment of COVID-19 patients (Table 2). Because of the drawback of poor bioavailability of curcumin, several strategies are applied to improve the oral bioavailability of curcumin in clinical trials containing nano-delivery systems (Hatamipour et al., 2019), with addition of adjuvants (Tabanelli et al., 2021) and new formulation (Flory et al., 2021). Inferiorly, the metabolic rate of curcumin is high in the intestine and liver, which causes its poor bioavailability. Extraordinarily, piperine is an inhibitor of glucuronidation in the liver and intestine (Kaur et al., 2018). Therefore, the combination of curcumin and 1% piperine is a potential option for the management of COVID-19 based on several properties including antiviral, anti-inflammatory, immunomodulatory, anti-fibrotic, and antioxidant effects (Miryan et al., 2021). In other clinical trials, results of non-COVID-19 treatment revealed that co-supplementation of curcumin/curcuminoids with 1% piperine could significantly improve the bioavailability (Panahi et al., 2015a; Shoba et al., 1998), and this formulation provides more antioxidant (Panahi et al., 2016a,b) and anti-inflammatory effects (Panahi et al., 2015b; Rahimnia et al., 2015). Different ayurvedic therapeutic agents (*C. Longa* L, green tea, and *Piper nigrum*) suppress the entry of virus into host cells, transmission of pathogens, and concurrently promote the immunity. Curcumin and piperine (1-piperoylpiperidine) interact with each other and form a π–π intermolecular complex which enhances the bioavailability of curcumin by inhibiting glucuronidation of curcumin in the liver (Kumar G. et al., 2021). The dose of curcumin from two clinical trial applications and one report for COVID-19 treatment is 1,000 mg/day curcumin plus 0.5–1% piperine (Askari et al., 2021; Pawar et al., 2021). When compared with the control group, the clinical results indicated that patients with the manifestation of mild, moderate, and severe symptoms orally receiving curcumin with piperine formulations as the adjuvant symptomatic therapy for COVID-19 infection have better clinical outcomes, and it can also reduce morbidity and mortality (Miryan et al., 2020).

**TABLE 2:** Clinical trial of curcumin on COVID-19 treatment of PubMed and ClinicalTrial.gov.

Formulation/design	Regimen	Administration	Patients (n)	Masking	Age (y)	Placebo (n)	Country	Authors
Nano-micellar gel	40 mg, four times/day	Oral	80	Double	19–69	40	Iran	Valizadeh et al. (2020)
Nano-micellar gel	40 mg, four times/day	Oral	40	Double	18–75	20	Iran	Hassaniazad et al., 2020*
Nano-micellar gel	80 mg, twice/day	Oral	60	Triple	18–65	30	Iran	Ahmadi et al. (2021)
Nano-micellar gel	40 mg, four times/day	Oral	40	Triple	18–75	20	Iran	Hassaniazad et al. (2021)
Nano-micellar gel	80 mg, twice/day	Oral	41	None	18–75	20	Iran	Saber-Moghaddam et al. (2021)
Nano-micellar gel	80 mg, thrice/day	Oral	60	None	18–75	30	Iran	Asadirad et al. (2022)
Nano-micellar gel	40 mg, four times/day	Oral	48	Double	30–65	24	Iran	Honarkar Shafie et al. (2022)
CurcuRougeTM	90 mg, twice/day	Oral	60	Double	65–75	30	Japan	Kishimoto et al. (2021)
Curcumin add 5 mg piperine	500 mg, twice/day	Oral	100	Double	20–75	50	Iran	Miryan et al., 2020*
Curcumin add 5 mg piperine	500 mg, thrice/day	Oral	100 in ICU)	Double	20–75	50	Iran	Askari et al., 2021*
Curcumin add 2.5 mg piperine	525 mg, twice/day	Oral	140	Double	>18	70	India	Pawar et al. (2021)
Artemisinin, boswellia, curcumin, vitamin C, and nanoparticle	Artemisinin 12/8.4 mg, curcumin 40/28 mg, boswellia 30/21 mg, and Vitamin C 120/84 mg, twice/day	Spray	50, 240, 252	Double	>18	16, 80, 84	Israel	NCT04382040*, NCT05037162*, NCT04802382*
Curcumin, quercetin, and vitamin D	Curcumin 42 mg, quercetin 65 mg, and vitamin D 90 units, four times/day	Oral	100	None	>18	50	Pakistan, Belgium	NCT05008003*
Curcumin, quercetin, and vitamin D	Curcumin 168 mg, quercetin 260 mg, and vitamin D 360 units, twice/day	Oral	50	None	>18	25	Belgium	NCT04844658*
* as the clinical trial application; ICU: intensive care unit.								

Furthermore, curcumin incorporated with a nano-delivery system can enhance water solubility, decrease degradation, control slow release, and increase absorptive efficiency (Chen et al., 2020; Li et al., 2020; Quispe et al., 2021), thereby increasing circulation time, pharmacokinetics, and biodistribution. Cumulative evidence unveiled that encapsulated curcumin with a nano-delivery system has more significant effects than native curcumin in the clinical trials for the treatment in metabolic syndrome to decrease triglyceride levels in serum (Bateni et al., 2021); in coronary elective angioplasty treatment to improve total cholesterol, triglycerides, and antioxidative capacity (Helli et al., 2021); and in knee osteoarthritis treatment to improve the severity of symptoms (Hashemzadeh et al., 2020). Curcumin is re-proposed as a potential antiviral key for the treatment of SARS-CoV-2 based on its relation to the infection pathways. Moreover, the use of curcumin-loaded nanocarriers for increasing its bioavailability and therapeutic efficiency was highlighted in several clinical trials (Dourado et al., 2021; Hassaniazad et al., 2021; Tahmasebi et al., 2021a; Tahmasebi et al., 2021b; Valizadeh et al., 2020; Hassaniazad et al., 2020; Ahmadi et al., 2021; Saber-Moghaddam et al., 2021; Asadirad et al., 2022; Honarkar Shafie, et al., 2022). Additionally, the potential of the nanostructured systems and their synergistic action with curcumin on the molecular targets for viral infections have been explored (Hassaniazad et al., 2020). One report illustrates that the administration of nano-curcumin can accelerate recovery from the acute inflammatory phase of COVID-19 by mediating inflammatory immune responses (decrease in serum IFN-γ and IL-17, TBX21 mRNA; increase in serum IL-4 and TGF-β, FOXP3 mRNA) (Ahmadi et al., 2021). COVID-19 patients receiving nano-curcumin supplements could significantly increase the O_2_ saturation and decrease the scores of the Wisconsin Upper Respiratory System Survey (WURSS-24) in the third domain, fourth domain, and total score, indicating nano-curcumin supplementation could help decrease hypoxia and moderate the symptoms induced by SARS-CoV-2 infection (Honarkar Shafie, et al., 2022). After nano-curcumin treatment, a significant reduction in the frequency of Th17 cells, downregulation of Th17 cell-related factors, and decreased levels of Th17 cell-related cytokines were found in mild and severe COVID-19 patients, implying that curcumin could be a potential modulatory compound in improving the patient’s inflammatory condition (Hassaniazad et al., 2020; Saber-Moghaddam et al., 2021). In all these clinical trials of COVID-19 treatment, nano-micelle curcumin was applied with the dose of 160 or 240 mg/day, and the dosage was lower than that of native curcumin treatment. Positively, hospitalized COVID-19 patients with oral administration of nano-curcumin formulation can modulate the inflammatory cytokine expression and secretion, such as IFN-γ, IL-1β, IL-6, and TNF-α (Kishimoto et al., 2021; Asadirad et al., 2022); regulate inflammatory immune responses to accelerate the recovery of the acute inflammatory phase; and shorten total recovery time (Ahmadi et al., 2021; Saber-Moghaddam et al., 2021). Additionally, oral spray with nano-curcumin formulation was developed and has entered phase III clinical trial for COVID-19 treatment in Israel (NCT04382040, NCT05037162, NCT04802382). Moreover, a new modified starch–curcumin formulation could increase curcumin absorption and distribution in blood circulation over 150 times compared to oral administration of native curcumin. This result showed this can significantly improve the anti-inflammatory effects and reduce the neutrophil/lymphocyte ratio with the administration of lower dose (Kishimoto et al., 2021).

Clinical trials of curcumin for COVID-19 treatment from PubMed and ClinicalTrial.gov are listed in Table 2. There are some points worth noting from all those clinical trials with curcumin formulations as the co-supplementation in COVID-19 treatment. As the sample size of those clinical trials was too small, their results cannot be representative and promising. Within 2 years from 2019, the mutants of SARS-CoV-2 (S^m^) varied and increased, and the outbreak of the mutated strands was faster; consequently, patients diagnosed with COVID-19 were infected by different viral variants with different clinical symptoms, severity, and mortality. These several viral variants of COVID-19 spread simultaneously around the world, but most clinical trials with curcumin applications in COVID-19 treatment did not easily establish the sample criteria for the patient’s viral mutants. There some pieces of evidence show that curcumin has a significant inhibitory effect on cytochrome P450 (CYP450) enzymes (Mashayekhi-Sardoo et al., 2021; Sharma et al., 2022), which play important roles in the phase 1 metabolism of several clinical drugs, such as steroids (Zhao N. et al., 2021). Generally, steroids are widely used for anti-inflammation in the clinical treatment of COVID-19 (Lin et al., 2021). Therefore, the safety of curcumin or curcuminoids was approved by the U.S. FDA, but the inhibitory effect of curcumin on CYP450 isoenzymes still needs more studies to investigate the interaction between curcumin and clinical drugs for treating COVID-19.

### Promises and Perils of Curcuminoids on the Remedy of COVID-19

**FIGURE 2:**
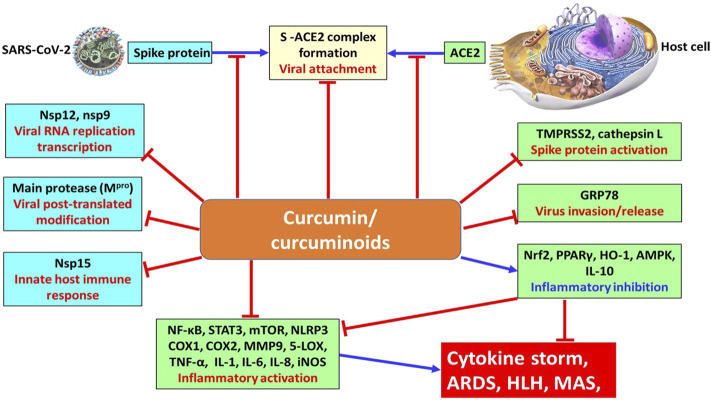
Underlying mechanism of curcumin and curcuminoids on the viral protein and host cell proteins for combating the COVID-19 pandemic. stimulation; inhibition.

Curcumin attenuates oxidative stress and inflammation and regulates inflammatory and pro-inflammatory pathways associated with most chronic diseases (Quiñonez-Flores et al., 2016), and it presents future perspectives regarding the usage of curcumin as an immunomodulatory drug. Paradoxically, curcumin (along with many other dietary polyphenols), as abovementioned, can target multiple organs or cell lineages without a known receptor or a defined target. This, along with other chemical features of curcumin, has generated enormous difficulties in the detailed dissection of mechanistic pathways underlying its biomedical functions. Indeed, these features have been causing some confusion among drug developers and have been heavily debated recently (Jin et al., 2018; Heger 2017; Nelson et al., 2017). Taken together, the pharmaceutical potential of curcumin and curcuminoids in the remedy of COVID-19 is depicted in Figure 2. The underlying mechanisms of curcumin and curcuminoids on the viral protein and host cell proteins in combating COVID-19 infection are also illustrated, including proposed signaling pathways of stimulation and inhibition from both viral and host cellular sites. Recently, with the awareness of the immunomodulatory properties of curcumin, curcumin was successfully added to different food matrices to formulate functional foods that can improve human body immunity to abate the outbreak of SARS-CoV-2 (reviewed in Tripathy et al., 2021). Further investigation is needed to unequivocally determine whether curcumin might provide a therapeutic benefit in various diseases, particularly COVID-19. The utilization of purified active compounds like curcumin at higher than normal doses warrants additional scrutiny, including an accurate determination of the appropriate dose, dosing regimen, duration of treatment, and further clarification of the mechanism(s) of action as it pertains to viral infection. With regard to the treatment of COVID-19 infection or any other viral infection disease in which curcumin is administered *via* the oral route, another important issue concerns the increase in the bioavailability of curcumin. Despite its efficacy and safety, the therapeutic potential of curcumin is indeed still debated due to relatively poor bioavailability in humans including its poor solubility in water, chemical instability, and a low pharmacokinetic profile (Anand et al., 2007). Importantly, the oral bioavailability of curcumin is low due to a relatively low absorption by the small intestine coupled with an extensive reductive and conjugative metabolism in the liver and elimination through the gall bladder. Moreover, although curcumin is orally administered at a high dose, only a small quantity is detected in the blood plasma that is rapidly metabolized and excreted via faces and urine (Niu et al., 2012). Aforementioned limitations have restricted curcumin’s therapeutic effectiveness in treating human diseases. Numerous studies have been conducted under heat and pressure with various formulations (amorphous) (Tran et al., 2019; Sunagawa et al., 2021) to increase the aqueous solubility and bioavailability of curcumin, which demonstrated that curcumin supplemented with nanoparticles of active substances or nano-formulations had enhanced bioavailability, controlled release, and increased stability. Additionally, lipid-based delivery systems and the encapsulation are mainly used for the enrichment of food products with health-promoting compounds. Notably, the delivery systems of curcumin (such as particles, micelles, emulsions, and liposomes) have a demonstrably positive effect on augment bioavailability (Chebl et al., 2017; Guerrero et al., 2018; Tan et al., 2018).

## Conclusion

Curcumin is a bioactive phytochemical that can be utilized as a nutraceutical or pharmaceutical in functional foods, supplements, and medicines. There are several limitations of curcumin use like low solubility and fast metabolism which restrict its absorption in the gastrointestinal tract and lead to poor oral bioavailability. Curcumin has multiple effects on the antioxidative stress, anti-inflammation, antiviral infections, but it has some inhibitory effects on the drug metabolism which need to be further clarified. To overcome these limitations, various curcumin formulations such as encapsulation in edible nanoparticles or microparticles to enhance its water dispersibility, chemical stability, and bioavailability are applied. Clinical trials of curcumin indicate safety, tolerability, and non-toxicity. However, the efficacy is questionable because of the small number of patients enrolled in each study. The challenges concerning research on curcumin’s health benefits are given as follows: clarifying the relationship between curcumin’s health benefits and the immunomodulation particularly in treatment of COVID-19 and conducting further human trials in which multiple research groups use the same samples and conditions. For the application of curcumin in COVID-19 treatment, further studies are still needed to optimize or improve its bioavailability following oral administration and to explore the degree of influence or interference of curcumin on clinical drugs metabolism. Moreover, this study after re-evaluation can afford a favorable application or approach of curcumin with evidence based on the drug discovery and development, pharmacology, functional foods, and nutraceuticals for effectively fighting COVID-19. Overall, curcumin is a promising ingredient of novel functional foods with protective efficacy in preventing or reducing the manifestation or complications of COVID-19 infection.
